# Tension pneumothorax and life saving diaphragmatic rupture: a case report and review of the literature

**DOI:** 10.1186/1749-7922-6-23

**Published:** 2011-08-01

**Authors:** Sylvain AA Pilate, Stefaan De Clercq

**Affiliations:** 1 Department of emergency, University hospital Antwerp, Wilrijkstraat 10, 2650 Edegem, Belgium; 2 Department of surgery, ZNA Stuivenberg - Erasmus, Antwerp, Belgium

**Keywords:** Tension pneumothorax, diaphragmatic rupture, diaphragmatic hernia, fecopneumothorax

## Abstract

A tension pneumothorax is a known life-threatening condition which requires a needle decompression. A diaphragmatic rupture is a relatively rare injury and is difficult to diagnose. A combination of a tension pneumothorax in presence of an ipsilateral diaphragmatic rupture can be called life-saving since the air in the pleural space is able to escape to the abdomen. The diagnosis of a diaphragmatic rupture by computed tomography or even by laparo- or thorascopy is crucial. Surgical repair should always be undertaken because the rupture will not close spontaneously and the risk of herniation of intra-abdominal organs to the pleural space will remain. In presence of a chest tube on suction, iatrogenic migration or even perforation of these organs can occur.

## Background

We describe a patient who presented with a traumatic left tension pneumothorax secondary to rib fractures. A computed tomography also showed a posterior left diaphragmatic rupture. We report a conservative approach with chest tubes that led to iatrogenic colonic perforation above the diaphragm after one week, thus creating a fecopneumothorax. A review is made on the diagnosis and treatment of post-traumatic tension pneumothorax with concomitant diaphragmatic rupture. We also review the pitfalls of the diagnosis of diaphragmatic ruptures.

## Case presentation

**Figure 1:**
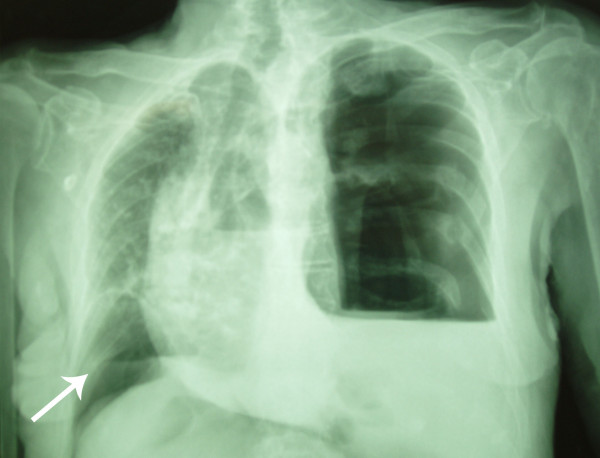
**Initial chest x-ray showing a left tension pneumothorax with shift of the mediastinum to the right, pleural effusion left, basal dorsolateral rib fractures**. There's also air visible under the right diaphragm (arrow).

**Figure 2:**
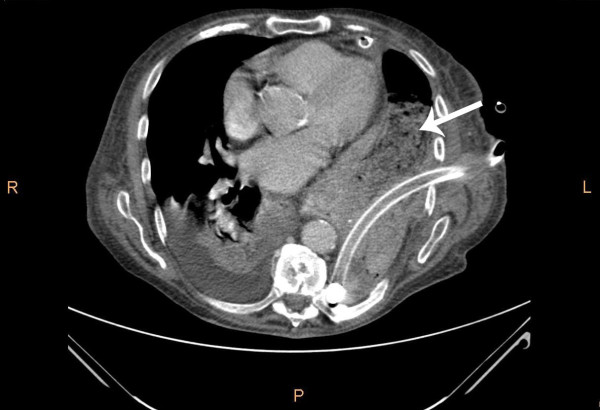
**Computed tomography on the seventh day showing intrathoracic presence of bowel (colon transversum) with feces (arrow) and a basal chest tube**.

**Figure 3:**
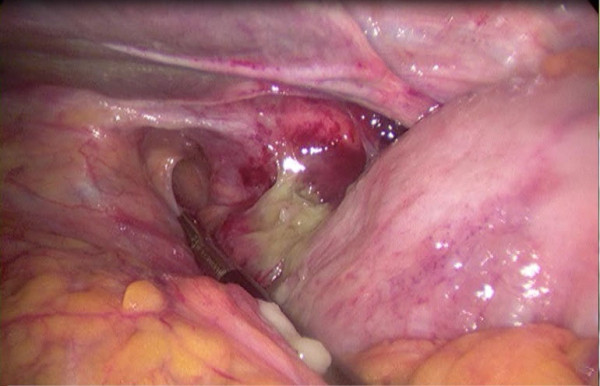
**Peroperative picture: left posterior diaphragmatic rupture**.

**Figure 4:**
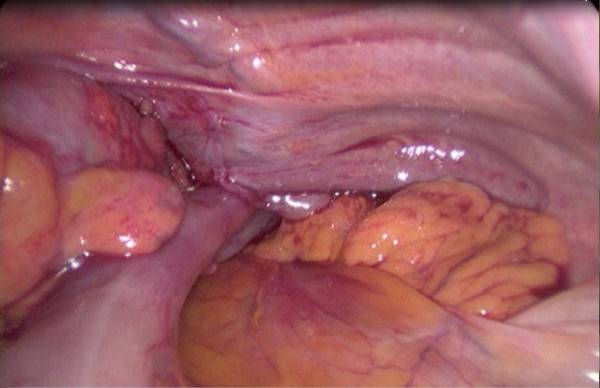
**Peroperative picture: colon transversum disappearing trough the diaphragmatic defect**.

A 92-year-old man was referred to the emergency department by his general practitioner because of suspicion of pneumonia. The patient reported increasing dyspnoea and bilateral pain at the thoracic base. Four weeks earlier he fell from the stairs and since then he suffered from mid-dorsal back pain. Physical examination of the lungs revealed tachypnoea, decreased breath sounds on the left side and unequal chest rise. Heart auscultation demonstrated regular rate tachycardia (110 bpm). The jugular venous pressure was raised. Abdominal examination showed a distended abdomen with hypoperistalsis, but no tenderness. On a chest x-ray a left tension pneumothorax was seen with pleural effusion on the left side and three recent basal dorsolateral rib fractures. Surprisingly a pneumoperitoneum was also visible on the chest x-ray (Figure 1). Needle decompression was immediately executed. Subsequently an apical chest tube was inserted on the left side and approximately 500 ml of serous and bloody fluid was drained. A computed tomography was made in search of the origin of intra-abdominal air. A left posterolateral diaphragmatic rupture was found. In respect to the patient's age a conservative approach was chosen. He was admitted to the intensive care unit and a second basal chest tube was inserted on the left side and broad spectrum antibiotics were administered. The chest tubes were kept on suction (-10 cm H2O) to accelerate the rate of healing. On the seventh day brown liquid was observed from the basal chest tube. A new computed tomography was performed and this showed herniation of the transverse colon through the hernia defect in the left diaphragm (Figure 2). The basal chest tube had perforated the colon, thus creating a left fecopneumothorax. A laparoscopic repair was planned. During this procedure the herniated and perforated part of the colon was removed, a transdiaphragmatic lavage was undertaken and the omentum was used to close the diaphragmatic defect (Figures 3 and 4). A mesh or sutures were not used since the abdomen was contaminated with feces. The 92-year-old-patient deceased on the fourth post-operative day due to respiratory insufficiency. Both the patient and family were in consent for abstinence from further invasive therapy.

## Discussion

A tension pneumothorax is the accumulation of air causing a pressure rise in the pleural space, generated by a unidirectional valve mechanism. The diagnosis is said to be clinical since it results in a life-threatening condition. Emergent needle decompression should be carried out before confirmation by chest x-ray when the patient is haemodynamic instable. The incidence of diaphragmatic injury among patients with blunt thoracic and abdominal trauma is about 3%-5% [1]. In this case we suspect that the left diaphragmatic injury resulted from the patient's fall from the stairs four weeks before his arrival at the emergency department. It is true that most diaphragmatic ruptures are due to high speed traffic accidents, but smaller accidents like a fall can cause the same type of injury [2]. Other etiologies might be an earlier trauma or a congenital posterolateral hernia (Bochdalek). The interval between diaphragmatic injury and the onset of symptoms can range from several weeks to years [3]. Left-sided rupture occurs approximately twice as often as right sided, due to protection of the liver [4]. When a traumatic diaphragmatic rupture is suspected a chest radiograph should be obtained because it remains the most sensitive method for diagnosis [5]. A computed tomography may show a discontinuity of the diaphragm, but it is not 100% sensitive. Herniation of intra-abdominal organs above the diaphragm is a possible complication of a diaphragmatic rupture. Surgical repair is necessary because the rupture will not close spontaneously. An undiagnosed or unrepaired diaphragmatic rupture can cause future hernation of intra-abdominal organs. Early diagnosis is crucial which was proven in a retrospective study with diaphragmatic herniation after penetrating trauma. The mortality rate in the group with early presentation was 3% compared to 25% in the group with delayed presentation (with a median of 27 months) [6]. A fecopneumothorax or a gastrothorax may rarely occur and may mimick the clinical presentation of a tension pneumothorax [3,7].

In this case the tension pneumothorax was secondary to rib fractures. The dorsolateral rib fractures were pointing towards the left lung. The hypothesis that the initial tension pneumothorax was a tension fecopneumothorax due to earlier colonic perforation above the diaphragmatic hernia was not withheld because of absence of feces or bacterial growth in the initial drainage fluid. A tension fecopneumothorax is a very rare identity and so far only 12 case reports have been published [8,9]. The perforation of the transverse colon was due to prolonged suction on the chest tube thus causing adherence and perforation of the herniated colon, resulting in a fecopneumothorax. As proven in this case a chest tube under prolonged suction might create an iatrogenic herniation of intra-abdominal organs and even perforation when a diaphragmatic rupture is present.

## Conclusion

In this case the presentation of the tension pneumothorax was subacute because the air was able to escape through the diaphragmatic rupture towards the peritoneum. A tension pneumothorax in presence of an ipsilateral diaphragmatic rupture can be called a life-saving combination. Unfortunately this diaphragmatic defect led to colonic herniation after one week thus allowing a chest tube to perforate the colon through suction. When a traumatic tension pneumothorax is clinically suspected a needle decompression should be performed. In the absence of haemodynamic compromise, it is prudent to wait for the results of an emergent chest x-ray prior to intervention. Afterwards a standard chest radiograph helps to look for signs of diaphragmatic herniation: elevation of the hemidiaphragm or the presence of bowel or stomach in the chest. A nasogastric tube can be seen above the diaphragm in herniation of the stomach. When a diaphragmatic rupture is suspected a laparoscopy or thoracosopy should be performed even with a negative computed tomography. A cautious approach is advised because a laparoscopy undertaken on a patient with a diaphragmatic rupture can lead to an iatrogenic tension pneumothorax. A diaphragmatic rupture must be repaired in presence of chest tubes as suction might cause iatrogenic herniation of intra-abdominal organs leading to perforation.

## Consent

Written informed consent was obtained from the the patient's relative for publication of this case report and any accompanying images. A copy of the written consent is available for review by the Editor-in-Chief of this journal

## Competing interests

The authors declare that they have no competing interests.

## Authors' contributions

SP drafted the manuscript. SDC made substantial revisions. Both authors have revised, read and approved the article.
